# Practical Departments

**Published:** 1905-07-01

**Authors:** 


### PRACTICAL DEPARTMENTS.

### THE BOSTEL FIRE.

The Coalbrookdale Co., of Rathbone Place, Oxford Street, W., and Coalbrookdale, Shropshire, who are well known as makers of apparatus involving iron and metal work of all kinds, are manufacturing the above. It is another attempt to solve the problem involved in heating the air of wards and rooms generally in an efficient manner at a low cost for coal, while preserving the cheerful appearance of the bright coal fire that is so dear to the average Englishman. The apparatus is a grate constructed on special lines. In place of the usual straight horizontal bars, on which the coals lie, with the curved horizontal bars enclosing them in front, the two sets of bars are made into one with the back, and assume the form of a basket, or a grate with only bottom bars, these being curved so as to take the place of both front and bottom bars. In addition to this, the fire basket is movable, tilts up and down on trunnions, and can easily be lifted out when necessary. When the fire basket of the apparatus is pushed into the vertical position, there is a free draught up the chimney, but when it is allowed to fall forward the draught is checked, as by a damper, the back slab being inclined forwards, and so partly closing the chimney space. The fire-basket and the back slab are arranged to be moved up or down together. When they are down, the ordinary position for regular use, the fire-bars rest in a space provided for them, above the ash-pit, which may be below the hearth, out of sight, the front of the fire-bars resting on the front portion of the hearth. The slab forming the back is then inclined forwards, and the draught is only that which passes over the coals and up the chimney, past the back slab. In this position, it is claimed, the combustion is very slow, and the whole of the heat liberated by the combustion of the coal is thrown out into the room, the back slab acting as a reflector for the heat rays as well as a check for the draught. The stove, it is estimated, will burn for eight hours with one charge of coal, which is $15\frac{1}{2}$ lb. of ordinary coal, without any special attention, and ordinary coal, or anthracite, can be used. The fire itself, it will be understood, when burning normally, is usually sunk in the ash-box. For lighting the fire—a matter that has given trouble with some grates which are sunk beneath the level of the hearth—the fire-bars and the back slab are tilted backwards, so that the back slab assumes the vertical position, allowing a free draught up the chimney, and the under sides of the grate-bars are exposed to the entering air, they assuming an angle of about 45 degrees with the vertical. The grate is held in this position till the coals have become thoroughly incandescent, when the grate and back slab are allowed to fall gently into their normal position. It usually takes from 15 to 20 minutes to accomplish this. If the fire burns low, the same process is adopted, after fresh coals have been placed on the fire. The process of tilting the grate and its back, it will be seen, is equivalent to the operation of putting an iron plate in front of the fire to draw it up, or of the dangerous practice, unfortunately so common, of propping a newspaper in front. In either case, the presence of the iron plate or the newspaper directs the air that enters the chimney through the coals in the grate, and tilting the Bostel grate does the same. It is claimed for the Bostel fire, amongst other virtues, that the air on the floor of the room is not so cold, and therefore one's feet do not become cold in a room warmed by one of the Bostel stoves as with the ordinary fire, where a draught is taken at the floor line. The fire can be fixed level with the hearth, below the hearth, or above the hearth as convenient, and is also applicable to stoves fixed in the middle of the room, with flues leading to chimneys at the side.

